# Exploring the theory, barriers and enablers for patient and public involvement across health, social care and patient safety: a systematic review of reviews

**DOI:** 10.1186/s12961-020-00644-3

**Published:** 2021-01-20

**Authors:** Josephine Ocloo, Sara Garfield, Bryony Dean Franklin, Shoba Dawson

**Affiliations:** 1 Centre for Implementation Science, Health Services, Population and Research Department, Institute of Psychiatry, Psychology & Neuroscience (IoPPN), King’s College London, UK; 2 National Institute for Health Research (NIHR) Applied Research Collaboration South London (NIHR ARC South London) At King’s College Hospital NHS Foundation Trust, London, UK; 3 Imperial College Healthcare NHS Trust, London, UK; 4 University College London School of Pharmacy, London, UK; 5 Centre for Academic Primary Care, Bristol Medical School, Population Health Sciences, University of Bristol, Bristol, UK

**Keywords:** Systematic review of reviews, Patient and public involvement, Theory, Barriers and enablers, Health, Social care and patient safety

## Abstract

**Background:**

The emergence of patient and public involvement (PPI) in healthcare in the UK can be traced as far back as the 1970s. More recently, campaigns by harmed patients have led to a renewed focus on strengthening PPI. There is a growing awareness of the benefits of PPI in research as well as a need to address power inequities and a lack of diversity and inclusion. This review was undertaken to look at evidence for theories, barriers and enablers in PPI across health, social care and patient safety that could be used to strengthen PPI and address a perceived knowledge and theory gap with PPI in patient safety.

**Methods:**

We searched MEDLINE, EMBASE and PsycINFO from inception to August 2018, using both MeSH and free-text terms to identify published empirical literature. Protocols in PROSPERO were also searched to identify any systematic reviews in progress. The extracted information was analysed using a narrative approach, which synthesises data using a descriptive method.

**Results:**

Forty-two reviews were identified and grouped by key outcomes. Twenty-two papers mentioned theory in some form, 31 mentioned equality and diversity (although with no theory mentioned in this area), and only 19 cited equality and diversity as a barrier or enabler. Thirty-four reviews identified barriers and enablers at different organisational levels: personal/individual; attitudes; health professional; roles and expectations; knowledge, information and communication; financing and resourcing; training; general support; recruitment and representation, PPI methods and working with communities and addressing power dynamics.

**Conclusions:**

The review findings suggest that a commitment to PPI and partnership working is dependent on taking a whole system approach. This needs to consider the complex individual and organisational enablers and constraints to this process and address imbalances of power experienced by different groups. Addressing equality and diversity and use of a theory-driven approach to guide PPI are neglected areas. The long tradition of involvement across health and social care can provide considerable expertise in thinking about ways to strengthen approaches to PPI. This is especially important in patient safety, with a much newer tradition of developing PPI than other areas of healthcare.

## Introduction:

The importance of involving patients, service users, carers and the public in the UK in health and social care and research has grown significantly in recent decades [1–4]. These developments have been linked to a growing recognition of the benefits of involving patients, their families and the public in contributing to research partnerships [5] and in making changes to service delivery and patient outcomes [6-8] as well as a growth in policy initiatives around citizenship, democracy and rights [9]. The idea of partnership working with patients and the public has also gained more prominence as a result of serious clinical and service failings in the UK [10, 11] and internationally [12-14]. These initiatives have often been driven by the campaigns of patients who have experienced harm and their relatives, leading to a new focus on PPI and its importance in improving quality and safety within healthcare.

In the United Kingdom, a commitment to patient and public involvement (PPI) in healthcare is enshrined in key legislation. This is covered by the Health and Social Care Act [15], the NHS Constitution [16] and the duty by NHS England (s13Q of the National Health Service Act 2006 as amended by the Health and Social Care Act 2012), to properly involve patients and the public in its commissioning processes and decisions. The Equality Act 2010 also prohibits discrimination in public services on the basis of nine protected characteristics (age, disability, gender reassignment, marriage and civil partnership, pregnancy and maternity, race, religion or belief sex, sexual orientation). This legislation can be used to encourage more inclusive involvement processes.

In addition to the above, key regulations set out essential standards of quality and safety that people who use health and adult social care services have a right to expect [17]. These rights are underpinned by wider but complimentary policy approaches such as public and patient experience and engagement (PPE). PPE approaches aim to place people who use services at the centre of care, to understand their experience of services, to empower them to make decisions and to involve them in the design and delivery of care [18]. The NHS Next Stage Review [19] identified three strands of quality: patient experience, patient safety and clinical effectiveness. PPE approaches aim to ensure that patient experience sits as an equal partner in these three strands of quality [19]. Policy documents, such as the NHS Five Year Forward View and Next Steps on the NHS Five Year Forward View, also reinforce how the health service needs to change, arguing for a more engaged relationship with patients, carers and citizens to promote wellbeing and prevent ill health [20, 21]. PPI and PPIE approaches are by no means unique to the UK, and similar reforms in health and social care can be seen across a range of international settings [6, 22–25].

Despite these policy developments, there have been an increasing number of criticisms about the nature of PPI in practice. There is uncertainty about how to do it well, in ways that constitute genuine partnerships and which involve a diversity of patients and the public, rather than a few selected individuals [7, 8, 26, 27]. In the area of patient safety, the literature on PPI has been seen as dominated by a biomedical approach [28, 29], atheoretical [30], and not addressing power inequities and discrimination [31]. This has exposed PPI to criticisms of exclusivity and tokenism [32].

These issues are seen as leading to a narrow model of PPI that fails to empower patients and the public in the involvement process. This type of model is out of keeping with a wider literature on PPI in health and social care, which highlights the contested and bottom-up nature and drivers for involvement and the way in which various global health social movements have provided collective challenges to poor care and discriminatory or paternalistic services and medical policy and belief systems [33]. These drivers have led to the development of theory, methods and approaches, particularly within mental health, that have been used to develop wider models and methods of involving patients and the public, based upon co-production and partnership [34]. In patient safety, despite the campaigns by patients who have experienced harm and their relatives acting as a catalyst for the patient safety movement, there is evidence that lay members are struggling to influence decisions and are largely expected to work within existing systems in improving quality and safety [11, 30, 35]. Involvement at this level has therefore been criticised as providing little opportunity to influence decision-making processes in any depth, maintaining power differentials and the status quo.

This review was undertaken to explore theories, barriers and enablers in undertaking PPI across health social care and patient safety, to identify evidence that could be used to strengthen PPI and, in particular, to address a perceived knowledge and theory gap relating to PPI in patient safety. To address this, the review set out to identify systematic reviews of the published empirical literature, to address the research question: ‘What are the theories, barriers and enablers in undertaking patient and public involvement in health, social care and patient safety?’ This paper will highlight the key evidence identified and what specific factors appear to hinder or strengthen the involvement agenda, which in turn could also help further develop PPI in patient safety.

## Terminology

There is considerable confusion about the use of terminology in this area. A number of different terms are often used synonymously with involvement, such as engagement or participation, whilst the terms patients and the public are also used interchangeably with ‘citizen’, ‘consumer’, ‘layperson’ or service user. These conceptual differences have emerged from disparate traditions, social movements, policies and practices to describe the involvement process [36]. They have also been used to imply a greater or lesser level of involvement, power or influence in decision-making processes within an organisation. However, this language does not always reflect the underlying ethos of involvement activities [37]. In the absence of a consensus on terminology, we define involvement as an activity that is done ‘with’ or ‘by’ patients or members of the public rather than ‘to’, ‘about’ or ‘for’ them [38]. This definition reflects the fact that the involvement process has increasingly come to be seen as a process of partnership: *“…the active participation of patients, carers, community representatives, community groups and the public in how services are planned, delivered and evaluated. It is broader and deeper than traditional consultation. It involves the ongoing process of developing and sustaining constructive relationships, building strong, active partnerships and holding a meaningful dialogue with stakeholders” *[17]*.*

## Methods

An overview of systematic reviews was conducted and reported in accordance with the Preferred Reporting Items for Systematic Reviews and Meta-Analyses (PRISMA) guidelines. The review protocol was registered in PROSPERO CRD42017067848 and subsequently published [39].

### Search strategy

A comprehensive search strategy was used to include a combination of five main blocks of terms including and relating to public involvement (public, patient, carer, consumer, citizen, lay, service user, stakeholder, family, relative, survivor), type of involvement (involvement, collaboration, engagement, partnership, consultation, participation, user-led, consumer or patient panel, advisory board/group/panel), health and social care setting (health services, healthcare, social care, public health, mental health, etc.), patient safety (safe*, adverse safety (safe*, adverse event$, error*, etc.) and type of review (systematic, narrative, meta), using a combination of Medical Subject Headings (MeSH) terms and free text (see Additional file 1). Terms used to describe public involvement and types of involvement were similar to other studies [40, 41]. Several scoping exercises in different electronic databases were applied to maximise the sensitivity and specificity of the developed search strategy.

Three electronic bibliographic databases, MEDLINE, EMBASE and PsycINFO, were searched for potential studies from inception to August 2018. Protocols in the PROSPERO database were also searched to identify any relevant systematic reviews in progress. Forward and backward referencing of included systematic reviews supplemented the database searches to identify any further relevant systematic reviews.

### Criteria for inclusion and exclusion

Studies were included in this review if they fulfilled the following criteria:

### Inclusion criteria

1. Type of study: systematic reviews that focused on the concept of, or approaches to, PPI and/or PPE across patient safety, healthcare and social care.
2. Setting: any organisational setting (e.g., primary care, mental health, hospital, tertiary care, voluntary, etc.).
3. Type of involvement: reviews that focused largely on the collective level or what has also been referred to as public involvement (this literature generally relates to public involvement in strategic decisions in health services, e.g., in service improvement planning, and/or organisational design, and can cover various areas at a local or national level in governance, policy making, commissioning, monitoring, evaluation and research). The review did, however, look at some examples of involvement in direct care, but only where this related to activities to improve health, social care or patient safety and quality more widely. The literature covering public involvement is distinct from literature focusing on patient involvement, which refers more specifically to ‘the involvement of individual patients, together with health professionals, in making decisions (including shared decision making) about their own care' [42]. The review did not include this much wider and more substantial body of literature on aspects or proxies for patient involvement, or engagement in their own clinical treatment such as shared decision-making and patient centredness.
4. Study design: systematic reviews based on either published empirical studies (e.g., using quantitative, qualitative or mixed methods) or theoretical papers. Where papers included both a systematic review and an empirical study, we included data relating to the review if it was presented separately.

Articles that met any of the following criteria were excluded

1. Systematic reviews that did not have a specific focus on PPI at the collective service improvement level.
2. Empirical studies.
3. Non-systematic reviews.
4. Reviews that focused on PPI at the individual level in treatment and decision-making.
5. Reviews not written in English.

### Screening and data extraction

An EndNote library was used to combine and export the results of the searches from different databases. Duplicates were removed prior to the selection of studies. Study selection was completed in two stages. First, three reviewers (JO, SG, SD) independently screened titles and abstracts to identify eligible and relevant reviews. Subsequently, full texts of relevant reviews were screened and reviewed in full by the two reviewers (JO and SD) for eligibility. Inter-rater reliability was high for both abstract screening (kappa coefficient = 0.83) and full text screening (kappa coefficient = 0.77). Any disagreements were resolved through discussions and, where necessary, consultation with the fourth reviewer (BDF).

A data extraction form was devised in Microsoft Excel and piloted using 10% of the included reviews by two reviewers (JO and SD) and discussed and amended as necessary. With the remaining reviews, data extraction was undertaken by two reviewers (JO and SD), with discrepancies resolved by consensus or in consultation with two other reviewers (SG and BDF) where necessary. No substantial disagreements occurred. The following data for included reviews were extracted:

Review characteristics—authors, year, aims, setting, country of origin of included studies, health topic focus, participant characteristics including sample size, evidence of equality and diversity, definitions of PPI, methods of involvement, theories/frameworks and concepts used/defined, evidence of barriers and facilitators to involvement.

The reviews identified were first classified inductively into different types of papers covering patient safety, healthcare and education, healthcare policy and guideline development, healthcare general involvement, research and community engagement and participation. These papers were then analysed to identify the evidence on barriers and enablers, theory and equality and diversity.

#### Deviation from protocol

We proposed that we would extract data on the impact of PPI from the included reviews [39]. However, due to the large volume of data extracted this will be reported elsewhere.

### Critical appraisal

Quality appraisal of the systematic reviews was assessed using the Assessment of Methodological Quality of Systematic Reviews (AMSTAR 2) [43] tool. This instrument was developed empirically for documenting the methodological quality of systematic reviews, including randomised controlled trials and non-randomised studies. The tool was used in this study to determine whether eligible reviews met the minimum criteria based on quality. AMSTAR 2 consists of a 16-point critical appraisal tool with evaluation options of “Yes”, “Partially Yes” and “No”. As suggested by Shea and colleagues [43], critical domains (items 2, 4, 7, 9, 11, 13 and 15) were identified for evaluating the included reviews (Appendix 1).

Quality appraisal was conducted independently by two reviewers (JO and SD) for a 10% sample of included reviews, followed by discussion for any discrepancies. The remaining reviews were appraised by one reviewer (SD) and checked by a second reviewer (JO). The overall quality of the included reviews was rated as high (n = 11), moderate (n = 2), low (n = 13) or critically low (n = 16), based on the critical domains (where errors or biases would significantly affect the validity of the conclusions of included reviews. If a review was categorised as low quality, this does not necessarily mean that it did not contribute to the discussion of barriers, facilitators and theories of PPI, but that the evidence for critical domains that affect the validity of the review and its conclusions was more limited. Such reviews were therefore included, but not treated with high confidence. The summarised AMSTAR 2 scores are presented in Appendix 1.

## Data analysis

The evidence from the reviews was grouped into themes and analysed through an iterative and inductive process that cut across the 42 reviews. After constant discussion and reflection on the findings between two researchers (JO and SD), they were initially categorised under broad headings and then refined several times as the data were re-examined and new headings and subheadings emerged. From here on within the results we will use the term review(s) to mean systematic review(s).

## Results

**Fig. 1:**
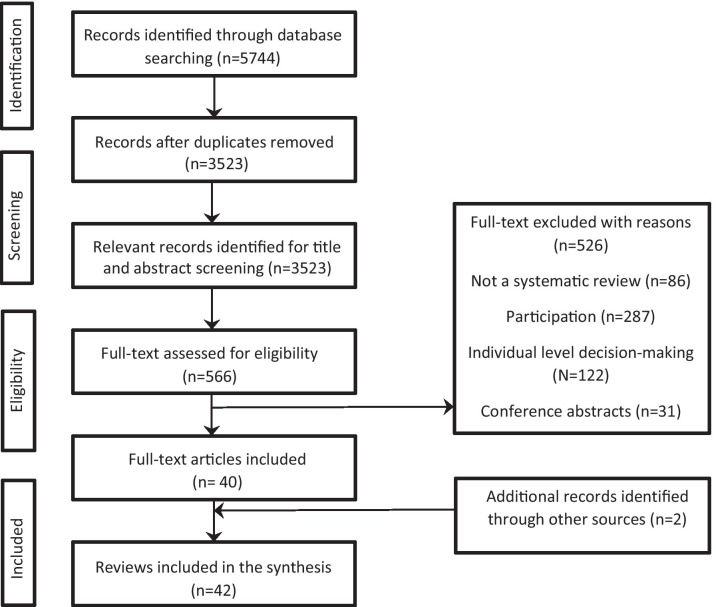
PRISMA flow diagram

As shown in Fig. 1 (below), the search strategy yielded 5744 articles. Following deduplication, 3523 articles were screened for titles and abstracts; of these, 566 were relevant for full-text screening. Forty-two reviews in total were identified as part of this review and are presented narratively grouped by our key outcomes. Twenty-two reviews mentioned theory in some form, 31 mentioned equality and diversity and 34 identified barriers and enablers. Summary of results are presented in Table 1.

**Table 1:** Summary of results

Results [types of reviews]	Patient safety	Healthcare & education	Healthcare policy and guideline development	Healthcare general/ involvement	Research	Community engagement/participation	Total
Total reviews	5 studies [44, 45, 46, 47, 48]	9 studies [49, 50]; [51, 52, 53, 54]; [55, 56, 57]	5 studies [58, 59, 60, 61, 62]	9 studies [63, 64, 65, 66, 67, 68, 69, 70, 71]	10 studies [72, 41, 73, 74, 75, 76, 77, 78, 79, 80]	4 studies [81, 82, 83, 84]	42
Theory [no of reviews that report theory]	4	Education & mental health [1] Education/general healthcare [3] Medical education [1]	2	6	2	3	22
Equality/Diversity [ED] [no of reviews that reported	4 [reported some data]	6 [reported some data]	4 [reported some data]	5	9	3	31
Barriers/Enablers [BE] [no of reviews that report BE]	4	8	3	7	8	4	34

## Barriers and enablers

A number of barriers and enablers were identified across 34 reviews. These were considered under key headings entitled: personal/individual factors, patient/relative involvement and attitudes, health professional relationships with patients, clarity of roles and expectations, knowledge, information and communication, financial compensation and resources, training, general support, power dynamics and organisational constraints, recruitment and representation, methods for involvement and recruitment and community approach. In some sections, there were papers that talked about the same factor as both a barrier and enabler. Therefore, where this occurred, we simply combined these factors and did not refer to them distinctly as barriers and enablers. Where we were able to differentiate the barriers and enablers, these were written up distinctly. These factors are also summarised in Table 2.

**Table 2:** Key barriers and enabler evidence

Barriers: health status, often age-related, wellness, self-confidence, diagnosis, proficiency in native language, privacy, embarrassment, patient consent, patient/family participation, relatives sometimes impede involvement, ethical issues in research, time factors, meeting paperwork/accessibility/timing, transportation, lay people feeling isolated/affected by the problem, capabilities being underestimated/contributions limited, little direct benefit/involvement fatigue Factors that could be either barriers or enablers: patients’ experiences/cognitive characteristics shape willingness to be involved in safety/lay people feeling subordinate to clinicians Enablers in safety: self-efficacy, feeling confident with error prevention/extraversion as a personality trait/attitudes about the fear/risk of errors. Relatives’ involvement in safety (self-administration of medication) in the home, (self-management of oral anticoagulant therapy) in hospital/supporting family members Enablers in education: self-reflection for users/nurse educators, patients as experts in their care, equal partnerships, increased involvement with mental health service users when well, excellent interpersonal/communication skills (which could also exclude people) Barriers identified by professionals/institutions: negative attitudes about patient contribution, consumer identity questioned, concerns about consumers’ mental health/unpredictable input/student safety Factors that could be either barriers or enablers: Health professionals play an important role in enabling patient involvement in patient safety: e.g., clinicians’ communication/listening, nurses’ positive attitudes/avoidance of negative reactions/acceptance of involvement/patients more able to challenge nurses than doctors Not all patients want/are able to participate in safety. Greater involvement in safety can be achieved by placing patients at the centre of healthcare. Managers can provide an appropriate work environment, through good communication, education/partnership working, role modelling to support involvement, patient feedback on safety/complaints, empowering patients to learn about their own health condition/report incidents Factors that could be either barriers or enablers: clear definitions/understanding about roles/definitions in PPI, goals of participation, experience/expertise required Enablers: specification of time commitment. In education/mental health, preparation of role/briefing/debriefing if partnership/equality to be promoted, including clarity of the academic role in supporting users, clarity when explaining research methods Barriers: Not all teachers/academics are convinced about the benefits of involvement, need for professional self-reflection, more information on teaching expectations, concerns about consumers revisiting painful experiences Factors that could be either barriers or enablers: provision of knowledge, clear information/communication positively associated with involvement in safety, access to information increases self-efficacy/risk perceptions increasing intention to act, not all patients are adequately informed Enablers: encouraging patients’ questions/clinicians to wash hands, posters/leaflets as reminders of information, professionals not reacting negatively to challenges, organisational processes can prevent nurses educating patients. Professional knowledge of patient education to promote patient autonomy, guidance for implementing patient/relative led escalation, effective/different methods of communication, adequate interaction time, dialogue, sharing of knowledge, collaborative working valuing users’ expertise Barriers: limited knowledge of involvement within education/professional institutions, use of technical language/processes/professional jargon, speed at which discussions take place, integrating patients’ experiential knowledge into evidence-based guidelines, emailing large documents rather than face to face meeting, health literacy, researchers’ unfamiliarity with consumer organisations, guidance for telling personal stories	Factors that could be either barriers or enablers: adequate funding/resources for involvement, disclosure of funds available, investment by universities to build engagement, time/cost of involvement, impact of financial incentives on recruitment, methods for collecting patients’ views time consuming/costly, staff time to support service users is costly, payment/remuneration for lay people, but could interfere with welfare benefits, further guidance needed with payments for mental health service users in education. Training for lay people is important [see specific examples in main findings section]. Support for lay people is a key requirement, see specific examples in main findings section] Enablers: researchers adopting a positive attitude towards involvement/fostering trust/mutual respect, being able to challenge health professionals, collaborative approaches to address power relations, self-reflection/co-publication, co-learning, involving patients at every stage/desired level of involvement Barriers: power imbalances with healthcare staff, lack of cultural drivers placing patients at the centre of safety/partnerships, hierarchical/elitist/paternalistic medical cultures create passive patients unwilling to engage with their safety, leaving patient concerns unresolved, professionals not relinquishing power/tokenism, resistance to patients’ participation, lack of involvement in key decisions/programmes, professionals feeling threatened by a reduction of influence/resources overwhelmed Barriers in education: emphasis on educators’ perspectives, lecturers’ positions, as gatekeepers to involvement, frightening experiences lead to nurses using defensive distancing strategies, professional fears about accountability, potential conflicts in professional/user agendas, professional fears about being blamed for deficiencies in mental health services More broadly barriers include frictions in relationships between professionals/lay members/leadership tensions, differences in priorities between researchers’/PPI members, suboptimal experiences of service users can impact research relationships Organisational barriers include: competing demands, staff shortages/staff turnover, high patient to nurse ratios, lack of staff buy-in, intervention complexity staff workload/time constraints, clinician fears about challenges to a medical-model. In education barriers include: the structure of teaching sessions, adherence to the rules of the academic institution regardless of implications for consumers, patronising culture/parentalism In community engagement/participation issues of power, privilege, discrimination, financial inequity, inaccessible communication, distrust and cynicism are barriers to peer partnership Factors that could be either barriers or enablers: There is a need for an organisational commitment/cultural shift if higher levels of involvement to happen, partnership strategies should be developed/tracked. Wider external drivers include the social, political, economic, geographic impact on community engagement or public health interventions, the influence of government policy/targets on health, a gap in the legal/political commitment to involvement Factors that could be either barriers or enablers: Concerns were raised about representativeness, lack of diverse perspectives/experiences, recruitment of appropriate participants seen as sensitive/time-consuming, high selection standards needed to address understanding of scientific data/but raises questions about representation, patient organisations may help recruitment but don’t cover many diseases, challenges exist collaborating with groups less willing to be constrained by research evidence Enablers: structures for reporting PPI to funders. In curriculum design/classroom-based activities [see examples in main findings section] Factors that could be either barriers or enablers: use of bilingual researchers, independent facilitation, embedding community engagement in the design of interventions. Community engagement processes can influence health outcomes: e.g., adequate time for community members/stakeholders to build relationships/agree on a ‘level playing field' [see results for more details] A need for adequate participant/provider skills training, administrative support. Greater satisfaction/effectiveness when meetings facilitated/held by community organisations than in clinics/health systems Transparency, collaboration, honest communication help address barriers to developing relevant community projects. Higher levels of peer engagement increased community involvement/trust, communication/impact. Barriers include: geography/physical location of project activities

## Personal/individual factors

In patient safety, people who were able to be involved, were generally willing to be involved [44]. Key barriers in this area included: a patient’s health status, which was often age-related [44, 47], wellness [65], self-confidence [63] and time to deal with diagnosis [65], patients’ lack of proficiency in the native language is a considerable barrier in error reduction [44, 46], issues of privacy [54], embarrassment [53, 54], patient consent [54], patient and family willingness to participate more generally in healthcare [65], involvement of patients’ relatives sometimes impedes patient involvement [47], the need to consider ethical issues in relation to confidentiality, anonymity and informed consent both on the part of service users and to protect other participants in research [73], time to be involved [41, 58, 63, 65, 73, 75] and to deal with the paperwork involved and what needs to be covered in meetings [65], accessibility and timing of meetings [65, 68], transportation [77], lay people feeling isolated or little affected by the problem in hand, their capabilities being underestimated or contributions limited [59], little direct personal benefit or involvement fatigue on the part of lay members [65].

## Patient/relative involvement and attitudes

Patients’ experiences and cognitive characteristics shape willingness to be involved in safety [44, 46–48]. Perception by lay people of their role and status as subordinate to that of clinicians [44, 47] acts as a barrier. Key enablers to involvement include self-efficacy, feeling confident and comfortable with error prevention, extraversion as a personality trait and attitudes about the fear or risk of errors increased willingness to act [44, 47, 48]. Relatives appeared to play an important role in safety, in the prevention of medication errors in the home (self-administration of medication), in hospital (self-management of oral anticoagulant therapy) [44] and in supporting family members by raising issues on their behalf [44].

In education, self-reflective experiences for both users and nurse educators were identified as enablers [57, 66]. In addition, a need was identified to recognise patients as experts in their care [66] and a need for a change in attitudes from paternalism towards equal partnerships [67, 69] and to valuing all involved [69]. In mental health, increased involvement with users when they were well helped develop more positive attitudes towards people with mental illness [57]; identifying patients with excellent interpersonal and communication skills was seen as a strategy for involvement but equally that it might exclude patients who might still be willing, or appropriate to act as teachers [55].

Lay people spoke about positive aspects of their involvement: such as enjoying the experience and believing it was worthwhile, talking and listening to students and gaining increased confidence [49]. They also had concerns about students’ negative attitudes towards their input in teaching and learning [49, 57], reported feeling vulnerable and exposed as a result of their contribution [49], perceived staff not to value lived experience [49, 58] and experienced voyeurism (staff wanting to know all about the consumer) [49].

Barriers identified by professionals and institutions included: negative attitudes about how patients could contribute [65] and lecturers who questioned who could call themselves a consumer, seeming to prefer consumers receiving treatment [49]. Lecturers raised concerns about consumers’ mental health and unpredictable input and students about their safety [49]. More generally in systems involving patients and relatives in the process of escalating in-hospital clinical deterioration, barriers identified for health-care staff included concerns that patients and families would summon a Rapid Response Team for frivolous or non-emergent reasons [64].

## Health professional relationships with patients

Health professionals play an important role in enabling patient involvement in patient safety [44, 46–48], for example, in clinicians’ ability to communicate, listen, encourage or instruct patients to ask questions or participate in specific actions [44, 48]. Professionals also need to be aware that not all patients want or are able to participate in safety [47]. Nurses’ positive attitudes, encouragement, support and education in safety practices and the avoidance of negative reactions and acceptance of involvement and questioning improve patients’ feelings of trust in their own ability [47]. Patients reported intervening more with nurses than doctors if challenges were required in the relationship [48].

More broadly, it is suggested that greater involvement can be achieved through placing patients and their safety at the centre of healthcare [46, 48]. Managers are important in providing an appropriate work environment to involve patients in safety and to support patient centredness, through good communication, education and partnership working, role modelling of patient involvement in harm prevention, support for patient feedback on safety and complaints and empowering patients to learn about their own health condition and to report incidents [47].

## Clarity of roles and expectations

Having clear definitions and understanding about roles was seen as important in PPI [41, 59, 73, 75]. Uncertainty existed amongst lay members about the goals of participation [58], their understanding and clarity about their role [41, 63, 68] and responsibilities [41, 77], and lay members had concerns about whether they had enough previous experience and expertise required for a PPI role [77]. Enablers included a well-defined outcome-focused presentation [63] and specification of the time commitment expected [49, 57, 59]. In the context of involvement in education and mental health, preparation of the role and briefing and debriefing was seen as essential if partnership and equality were going to be promoted [57]. This included clarity of the academic role in supporting users in a classroom setting and challenging students’ comments about unethical care and practice if necessary [57] and clarity when explaining research methods [72]. Barriers included not all teachers/academics being convinced about the benefits of involvement and worried it might threaten their role [49, 52]. The need for professional self-reflection was identified [52] and lay members thought staff provided limited information on teaching expectations [49]. There were also concerns from lecturers about consumers having to revisit painful experiences [54].

## Knowledge, information and communication

Provision of knowledge, clear information and communication was found to be positively associated with involvement in safety [44, 47, 48]. Access to information was found to increase self-efficacy and risk perceptions increasing intention to act [44] and ability to monitor and detect errors [47]. Not all patients were knowledgeable or felt adequately informed [47]. Enablers included simple visual reminders, encouraging patients to ask questions or tell clinicians to wash their hands [44] or posters and leaflets provided in patients’ rooms to remind them and their relatives of the information they received from the admitting nurse [64]. Professional education should include the importance of avoiding negative reactions to perceived ‘challenges’ and discouraging responses [47]. Organisational processes can also prevent nurses educating patients [47]. Professional knowledge of patient education to promote patient autonomy is important [66], as are guidance for implementing patient- and relative-led escalation [64], use of effective communication [66, 68, 73], adequate interaction time [63], use of different methods of communication [69] and dialogue [68], with information [73] and sharing of knowledge enabling the balance of power to be redistributed to allow collaborative working to occur [52, 69] and the valuing of users expertise [52].

Barriers include limited knowledge of and experience within education settings which can limit lay involvement [49], use of technical language/processes and professional jargon [54, 58–60, 63, 65, 68, 72, 77], speed at which discussions take place, difficulties with integrating patients’ experiential knowledge into otherwise evidence-based guidelines [58], lack of professional/institutional knowledge of involvement [65], sending large documents by email rather than having face-to-face meetings with lay members thereby limiting consensus [59], health literacy [65], researchers’ unfamiliarity with consumer organisations and their ways of working [63], more guidance and time to tell personal stories [53].

## Financial compensation and resources

Adequate funding and resources were identified as key [41, 58, 59, 65–69, 73, 75, 83], including disclosure of the funds available [59]. Significant investment is needed by Higher Education to build engagement into these institutions [51]. There was an identified need to address the time and cost implications of involvement [41, 58, 72, 75, 77]. The impact of financial incentives on recruitment was reported [73], and the use of indirect methods for collecting patients’ views (focus groups, self-administered questionnaires, or individual interviews) was seen as time-consuming and costly [60]. The cost of staff time to prepare, support and accompany service users was seen as not insignificant [51]. The process of producing recommendations was expensive and obtaining consensus took longer, and the priorities were deemed to be lower quality when scored when patients were involved [82].

The need for payment and remuneration to lay people was raised by many reviews [41, 51, 53–56, 59, 63, 65, 68, 72–74, 77]. However, payment could interfere with people’s welfare benefits [49, 57]. Payment to mental health service users in education was seen as about valuing user involvement, but required further guidance [57].

## Training

Training for lay people was mentioned in several reviews [53, 58–60, 63, 66–69, 74, 76, 78]. Examples included: skills development for lay people [63], induction, ongoing training [51, 54], seminars to assist with technical matters, critical appraisal skills [59], specialist medical facts [60], including from multiple sources: online, face-to-face mentoring, workshops being co-produced and covering areas such as equality and diversity, confidentiality and data protection, structure of healthcare organisations, clarity of role and orientation [68] as well as a tailored approach to match the aim, purpose and local context [69]. Training was also suggested for meeting chairs to enable them to ensure patients can deliver their input [59], including developing the confidence and competency of surgical healthcare professionals [76] and researchers to develop PPI [73, 76, 78]. In education various training strategies included: classroom-based homework using manuals or videos, moving learning from the classroom into patients’ homes and scheduling teaching to take place in the evening [55, 56]. However, there was a lack of clear guidelines as to what this training should constitute and how it should be evaluated [55]. It was also noted that there should be training or guidance for professionals in partnership working or joint decision-making [65].

## General support

Support for lay people (or lack of it) was identified as a key requirement [51, 53, 54, 57–60, 74], including before and after initiatives [69]. This included: emotional [51, 69], financial [51, 54] and practical support to do with timing of activities and venue [53, 63], appropriate setting, safe environment [63], time constraints and commitments of public members [68], support with telephone and e-mail [59], access to the internet and IT support, sending documents well ahead of meetings, having at least two lay members to support each other, public representatives having an identified team member’s contact details, being paired with a buddy/more experienced lay member, handovers between lay members [68], using lay people with previous experience [63], mentoring [59, 63, 68], a supportive chair, an analysis grid for knowledge synthesis, a welcome pack, assistance with complex scientific and technical issues, offering participants opportunities to interact with other or previous patient representatives, and involving a group of patients rather than a single patient [59].

Suggestions for use of a welcome and information pack [59, 63, 68] included: having information on the organisation, time commitment, remuneration [68], induction [63, 68], disqualification criteria, governance/public life standards/complaints procedure, meeting schedules, contact details, role of the board and lay members, and glossary of key terms/terminology [68]. Other support mechanisms included: use of an open working style and innovative culture [63], agreeing on principles of user-involvement, consultation at the planning stage to establish an environment of shared ownership, support and commitment [57], discussions on issues of confidentiality [49, 57], feedback to PPI contributors [41, 73, 75], building trust and relationships [77], and supportive interdisciplinary relationships [66]. Projects need high levels of commitment with user involvement and need to appoint a project worker to ensure success [49].

## Power dynamics and organisational constraints

Issues of power as enablers to involvement were identified in different ways in the reviews: at an individual level through researchers adopting a positive attitude towards involvement and fostering trust and mutual respect between parties [41, 73, 75] and through the ability to challenge health professionals where necessary [68]. Collaborative approaches were also found to address paternalism and power relations and help with self-reflection and co-publication [57]. This included building reciprocal relationships [75, 78], co-learning [78] and actively involving patients at every stage of the process [58, 59] and at patients’ desired level of involvement.

Barriers included power imbalances in relationships with healthcare staff [44, 47, 52, 54], a lack of cultural drivers to place patients at the centre of safety and partnership working [47], the hierarchical, elitist and paternalistic culture of the medical profession, creating passive recipients of medical expertise [44, 52] and creating patients unwilling to engage with their safety [44].

Patients were found to lose confidence and avoid future contact and cooperation if healthcare providers avoid partnership or leave concerns unresolved [47]. Difficulties were also found with professionals relinquishing power, tokenism [49, 65, 75] and resistance to patients’ participation [59] and involvement in key decisions and programmes. Professionals can also feel threatened by a possible reduction of influence [65], feeling their role will be undermined [46, 47] or resources overwhelmed.

In education there were a number of barriers in terms of power imbalances at the professional and organisational level [52, 54]. On a professional level, there was an emphasis on educators’ perspectives of consumer involvement [52, 57], power dynamics related to lecturers’ positions as gatekeepers to consumer involvement [52] and frightening experiences leading to nurses using defensive distancing strategies, reinforcing a ‘them and us’ culture [57]. Professionals also had concerns about ethics, e.g., users advocating something that would compromise professional accountability [52], potential conflicts that could arise over the boundaries of group discussions or different agendas or of being blamed for deficiencies in mental health services [49]. More generally there can be friction in relationships between professionals and lay members [72], leadership may be questioned on either side [65], differences in priorities can exist between researchers and PPI members [77], and their perspectives [59] and suboptimal experiences of service users can also affect future relationships with researchers [77].

At the organisational level, competing demands, staff shortages/staff turnover, high patient-to-nurse ratios, lack of staff buy-in and intervention complexity [46], staff workload, time constraints [47, 58, 59] and advice from expert patients which could be seen as a significant change from a medical model and viewed negatively by clinicians [65] were all barriers to involvement. In education, the structure of teaching sessions [49], adherence to the rules of the academic institution regardless of the implications for consumers and a patronising culture and parentalism were found [52]. In the area of community engagement and participation, issues of power, privilege, discrimination, financial inequity, accessible communication, distrust and cynicism were all identified as barriers to peer partnership [84].

There is a need for an organisational commitment to involvement [54, 69] and a cultural shift if higher levels of user involvement and partnership are to happen [52], with strategies developed, tracked and debated, with equal representation from consumers and academics/professionals [54]. Wider external drivers include the social, political, economic and geographic context and its impact on community engagement or public health interventions and the influence of externally imposed government policy and targets for achieving health [83] and a need to address a gap in a legal and political commitment to patient involvement as a right [65].

## Recruitment and representation

A number of concerns were raised about issues of representativeness and how and why people were chosen to be involved [49, 72] and lack of diverse perspectives, experiences, expectations and interests [73]. Recruitment of appropriate participants was seen as sensitive and time-consuming [63]; some reported difficulty in recruiting lay people, particularly those who were able to contribute significantly to the discussion among experts [59, 60]; others noted high selection standards for patient candidates (e.g., to address ability to understand scientific data), raising questions about representation [58]. Patient organisations may help recruit patient representatives who have an appropriate level of knowledge; however, for many diseases the number of patient organisations/self-help groups available is very low [60]. There are also challenges tof collaborating with consumer organisations who might be less willing to be constrained by research evidence [63].

Recruitment strategies identified included: inpatient or outpatient settings; existing training or ‘patients-as-teachers’ programmes or use of women’s health and educational consultants or university groups, self-help or service user or community groups, e.g., mental health services user groups, HIV support, women’s groups and outreach programmes, advertising in newsletters or through posters or through other patients, purposive recruitment of subjects including paramedics, degree students, nurses and midwives, clinical staff and qualified educators and use of innovative methods with donors [55, 56].

## Methods for involvement and recruitment

A number of reviews talked about using a combination and different methods for enabling involvement [53, 58–60], including structures for the reporting of PPI, identified as important for funders [76], for example in curriculum design and classroom-based activities (Delphi studies, service user reference groups, focus groups, discussion groups, curriculum design groups, consumer groups, strategic development groups). Curriculum delivery strategies included: involvement in classroom activities, practice-based interventions, skills workshops, the development of learning materials, the appointment of a service user to an academic post [53], using secure contracts [51], ensuring every academic module had at least one session delivered by service users and use of methods to reduce the bias of dominant opinion leaders in a group, such as individual interviews/broader surveys as well as individual participation [60]. In implementing teaching and learning methods involving service users, attention to debriefing, supervision, ethics and outcomes and a ‘right to refuse’ to answer questions for users was a useful ground rule. Professional accountability was also seen as something which should remain with lecturers on ethical grounds [57].

## Community approach

Key evidence identified in this area included: use of bilingual researchers [77], independent facilitation [72] and embedding community engagement as a key strategy in the design of interventions [83]. The process of engagement was thought to influence how well that activity ultimately impacted on health outcomes. This process included: adequate time for community members and other stakeholders to build relationships, so that they could agree on a ‘level playing field’ in terms of language, negotiation and collegial working skills, learning about funding sources and developing skills to bid for future sources of funding. There is also a need for adequate participant and provider skills training and the right amount and quality of administrative support to ensure smooth project running, activity timing, duration and frequency [83]. Greater satisfaction and effectiveness of deliberation were reported when meetings were facilitated and held by community organisations rather than in clinics or health systems [82].

Factors such as transparency, collaboration and honest communication helped address barriers and challenges to developing projects that were culturally and contextually relevant to communities [84]. Higher levels of peer engagement increased community involvement, trust, communication and impact. Barriers identified in this area included: local geography and the physical location of project activities, which were influential in determining the priorities to tackle and methods to use in a community assessment and level of participation in activities and availability of partners and power relations between stakeholders [81].

## Theory

Table 3 sets out the key evidence on theory from the review across the different categories of papers. Overall 22 reviews identified theory in some way.

**Table 3:** Theory and equality and diversity evidence

Patient Safety Theory Theory of planned behaviour [44, 48] Health belief model, status characteristics theory, role theory, safety culture, bio-medical model in decision-making [44] Picker's 'Eight Dimensions of Patient-Centred Care, patient activation measures and patient empowerment [46] General theory findings Clarifying theoretical models underlying the mechanism of patent engagement can help motivate successful interventions [46] Lack of a theoretical basis with no consideration of the intended mechanisms of interventions (how they will work), or where they will affect the ‘causal chain’ (where they will work) to improve safety [45]	Equality and Diversity [B and E stands for where reviews reported equality and diversity specifically as a barrier and enabler to PPI] Inconclusive evidence with respect to age, gender or education to claim these characteristics was a consistent factor predicting patients’ willingness or ability to engage with their own safety [44, B/E] The synthesis suggested that age is probably a confounding factor. ‘Commonly people who were able were willing’. Important barriers affecting ability are illness, which is often aged related, and ability to communicate in the native language. Findings suggest one underlying cause of inability to be involved actively, may be some age-related illnesses, rather than age itself [44][B] There is also some evidence that female, younger, higher educated patients and those who experienced errors or intensive episodes of care are more likely to have a positive attitude toward involvement in error-prevention strategies, but these patterns are inconsistent [48, E] Younger age of patient is a facilitator [E] and older age [B], patients’ primary language and cultural tendency to rely on physicians [46][B] Participants [outcomes] reported were limited and primarily English-speaking, literate adults and elderly patients. Targeting elderly patients to improve medication safety is seen as a reasonable strategy, as they are a group where comorbidity/poly-pharmacy are common. A need to evaluate the ability of other vulnerable groups: those with communication difficulties/low health literacy/understanding of how to be involved [45]
Healthcare & Education Transformative learning used in combination with user involvement [57] A five-step concept analysis of service user involvement in health and social care education is identified. The ‘ladder of involvement’ describes stages of ‘no involvement’; ‘limited involvement’; ‘growing involvement’; ‘collaboration’; ‘partnership’ [51] A ‘partnership’ approach is discussed, but not defined conceptually [54]. Concepts of expert patients and patient voice, collaboration and partnership and an attempt to build a framework of attribute for involving patients in education were discussed [55] General theory findings There is terminological confusion with concepts such as ‘collaboration’ or ‘user involvement’ or talking about involvement at different levels. These concepts need further analysis and confusion can lead to conflict with stakeholders [52] Use of a framework with headings: Who? How? What? Where? is identified as a useful starting point to stimulate more careful consideration about the involvement of patients in medical education. This framework is seen as a useful starting point, but other studies indicate other issues need to be considered including: gaining informed consent and systems of support for involved consumers [54]	A number of reviews raised concerns about the representativeness of consumers [49, ED], [BE] A need to include people currently using services and in more acute stages of mental illness as there were concerns that service users were not representative of the wider experience of service use [50] Procedures regarding the process/preparation for involving users in the classroom are needed to promote partnership, involvement and equality [57, B] A conference was described as a means to consult a range of consumer groups to inform social work training. Community groups were asked to nominate representatives from diverse religious and ethnic groups to discuss how future doctors could best serve diverse communities [54] There was concern by lecturers that someone who was mentally ill would not be able to teach large groups of students and if they were, then they could not be considered representative of the core group of users, thus indicating a kind of ‘catch 22′ situation [54, B] Concerns were described about: wider involvement from all staff; strategies to avoid stigmatisation/discrimination that are not always effective; issues of inequality of power between service users and educationalists; attempts to redress power differentials that could be impacted by cultural barriers to its success [53] In medical education, some issues described on equality and diversity included students visiting patients living in deprived areas to raise awareness of disease and links to social deprivation and the need to ensure adequate representation of different patient groups in education [55] Attributes patients may need to possess to be involved, such as excellent interpersonal/communication skills, may exclude other groups such as elderly people, people belonging to ethnic minority groups and people for whom English is not their first language [55]
Healthcare Policy & Guideline Development A typology of public engagement mechanisms [59] General theory finding A considerable overlap exists between concepts and methods of public involvement, e.g., consultation through to partnership [61]	One study reported on various community projects targeting disadvantaged groups (youth, isolated families, ethnic minorities) to increase local access to care services [61] Criteria used for patient selection (i.e., representation, demographics) in different studies varied [60]. There were challenges to gathering a well-balanced group that represents different age, socioeconomic and ethnic groups and which consists of patients in different stages of the disease [60, B] One of the main challenges is the recruitment of patients or patient representatives who are able to contribute significantly to the discussion among experts. At the same time, they should be “legitimised” to represent the broad spectrum of patients with different socioeconomic and health characteristics and attitudes toward healthcare [60, B] Representation of patients and members of the public [59, B]. Professionals’ resistance to patients’ participation [59, B] Patients have difficulty following/assessing medical/technical jargon. Several authors pointed out that as a consequence the patients gave little input. This could lead to high selection standards for patient candidates (e.g., for a NICE clinical development group). If, however, only highly educated patient representatives are recruited, how representative is the patient input? [58, B]
Healthcare Generally and Involvement Gauvin’s conceptual framework 2010 to map the concepts related to patient and public involvement in health technology assessment (HTA) [63] Rodgers’ evolutionary method of analysis for concept development to re-examine the concept of partnership [66] 7 rationales for the involvement of people affected by cancer in research, policy and planning, and practice included: models for patient experience, personal empowerment, the autonomous patient, marketing, democracy, community and policy directives. These were seen to reflect ideologies of individualism and collectivism [67] 13 PPI principles were viewed theoretically as a principle taxonomy for those looking to work together [69] Conceptual or theoretical underpinning was scarce. Two studies used a theoretical argument. One argued for collective self-advocacy in balancing power differentials; the other drew upon social constructionism and post-modernism to challenge the professional narrative in mental health services. Most studies relied upon policy initiatives as their primary framework [71] General theory findings Conceptual/terminological confusion and challenges were identified to do with the term patient engagement [65]	Reference to equality in the context of sharing power in partnerships [66], reference to being ‘inclusive’ made under key principles for involvement [69, BE]. Approaches for working with communities included: reaching out to relevant communities, not expecting them to come to you [69, BE], training in the area of equal opportunities/equality and diversity [68, E], the need for a broad representation of individuals to be involved [65, 67], including those traditionally excluded [67], with a variety of health related experiences to ensure a responsive approach to their needs [65], it is also noted it is not conclusive whether socio-demographic factors influence the extent to which patients wish to be involved in treatment decision-making [67] Communication—Language, in terms of health literacy and especially with the use of technical terms, was a barrier to patient involvement [65, B]. Personal characteristics of patients like ethnicity, age and disease might lead to discrimination and therefore lower opportunities for involvement [65, B]
Health & Social Care Research 37 sources were identified describing frameworks/conceptualisations of Patient Service User Engagement (PSUE) converged into a synthesised framework comprising 3 broad phases of research (preparatory, execution and translational phases) [78] General theory finding Attempts were made to distinguish theoretical boundaries between service user involvement in research and other types of involvement, e.g., in own care/the delivery of care for a relative or in educational interventions. Current conceptualisations of service user involvement were seen as limited in terms of a hierarchy/continuum and not reflecting the fluid nature of involvement. A further conceptual weakness was there not being much known about what is ‘research data’ in a study and what is ‘service user involvement’ [73]	Issues of representation [72], cultural sensitivity [72][E] Patient Service User Research (PSUR) should consist of individuals or communities for whom the outcomes are of interest [E]. Many studies stated that, from the very beginning, researchers should see Patient and Service User Researchers (PSURs) as equal partners and consider them as a reliable component of the team rather than simply an additional variable or complication [E]; involvement of authority figures in the community was also helpful for buy-in [E]. Accessibility of dissemination approach to individuals with different abilities and preferences should consider language, terminology according to the target population and purpose of the publication [78] [B]. A review on (PSUR), noted several concerns, including inequality in the interactions between PSURs themselves [78, B] A few studies discussed how PPI increased community group membership and led to greater intercultural understanding by all parties involved in the research and created links to seldom-heard communities [41]. More generally working with the community seemed to provide some opportunities for working with a broader diversity of patient groups [41, E] Interviewers and employees who had been users all received training. Where applicable, this training was similar to that received by employees who had not been users of mental health services [74, E] Some studies showed the involvement of service users allowed researchers to have privileged access to a particular population or community groups; working with community leaders, patient networks or voluntary organisations were good approaches to engaging with seldom heard groups [73] It is a fundamental but often overlooked fact that service users have diverse perspectives, experiences, expectations and interests. Which service users are involved needs to be considered in relation to the purpose, aims and context of any proposed research. Such decisions should be explored, where possible with service users themselves [73, BE] No references were found regarding involving marginalised and minority groups in PPI in surgical research or on the use of PPI to integrate the opinions of marginalised and minority groups. Some studies discussed inclusion of feedback from study participants. This is not the same as active collaborative involvement where patients become partners in the research process [76] A few reviews also reported PPI contributor characteristics [80] and characteristics of those who influenced participation decisions in research [75] A common concern of researchers and patients was that patient engagement may become tokenistic (a false appearance of inclusiveness), resulting in a devaluated patients’ input [75, B] Disregard for cultural beliefs and language [B], use of bilingual researchers [BE, ED], explore culturally appropriate solutions [77, E]
Community engagement/participation in health Three synthesis ‘products’ on community engagement were identified: (i) theoretical meta-narratives indicating how community engagement is conceptualised; (ii) theory of change models that operationalised the theoretical meta-narratives; (iii) an overarching conceptual framework built on the findings from (i) and (ii). Two clear perspectives or ‘meta narratives’ emerged: a health services or ‘utilitarian’ perspective and a ‘social justice’ perspective Three hypothesised models were developed from this framework: classical or traditional, peer- or lay-delivered interventions [83] The review categorised types of community participation based on the continuum proposed by Popay et al.’s (2006) conceptual model. Theories such as Community Coalition Action Theory, Procedural Justice Theory, Community Capacity Framework, Kumpfer's model of leadership and partnership functioning, framework on social participation or citizenship and quality of care, people relationship-building framework, Communities of Care Model, Community-orientated Primary Care Model and Communities that Care (CTC) Model were commonly found. The concept of power was discussed with Bordieu’s (1986) theory of power discussed at great length. Overall, the review findings highlight that research in this topic area lacks robust study designs and theoretical underpinnings, in line with previous systematic reviews [81] Peer models were discussed in the light of 2 theoretical traditions: human-centred design (HCD) and action research [84] Key theory findings Peer models appeared to be successful when conducted within a theoretical model like HCD or action research that supports ongoing commitment to collaboration, co-learning, mutual leadership and shared decision-making as peers are more likely to feel valued when considered equal members of the project team [84]	In studies where patients developed their own care strategies, some of these strategies were considered effective as they enhanced care experiences or equitably improved outcomes and had greater impact in more socio-economically marginalised communities [82, E] Having diverse peer community participation in the planning and implementation of community-level surveys strengthened the communities’ commitment to using their results [82] Identified studies reporting that when patient groups assisted in recruitment and retention of research participants among marginalised populations, they produced greater recruitment numbers and retention [82][E] Equality and diversity issues were also identified as barriers to involvement including a racial divide leading to dissatisfaction amongst mainly white researchers who were being paid and predominantly African-American community members who were not. Gender inequality leading to skills being devalued in male-dominant settings. The socio-economic status of a community such as poor education, unemployment and poverty, affected rates of community participation. Communities often worked in partnerships with statutory or health authorities where the latter held power; therefore, they perceived participation as tokenistic [81, B] Peer community member participation in the planning and implementation of community-level surveys in 12 diverse communities in the state of Washington strengthened the communities’ commitment to using their results in the planning of environmental change strategies. Potential-related issues of power, privilege, discrimination, financial inequities, accessibility communication, distrust and cynicism can also contribute to peer partnership difficulties [84]

Some general theoretical findings identified across the reviews showed that in patient safety there is a need to clarify theoretical models underlying the mechanism of patent engagement to help motivate successful interventions [46]. There is a lack of a theoretical basis with no consideration of the intended mechanisms of interventions (how they will work) or where they will affect the ‘causal chain’ (where they will work) to improve safety [45]. In healthcare and education, the issue of terminological confusion was highlighted with concepts such as ‘collaboration’ or ‘user involvement’ or in talking about involvement at different levels [52]. In medical education, use of a framework with headings: Who? How? What? Where? was identified as a useful starting point to stimulate more careful consideration about the involvement of patients. Further issues that need to be considered include gaining informed consent and systems of support for involved consumers [54].

In the area of healthcare policy and guideline development, a considerable overlap was identified between concepts and methods of public involvement, e.g., consultation through to partnership [61]. More generally across healthcare, conceptual and terminological confusion was found and challenges to do with the term patient engagement [65]. In health and social care research, conceptualisations of service user involvement were seen as limited in terms of a hierarchy or continuum not reflecting the fluid nature of involvement. A further conceptual weakness was considered to be that not much is known about what is ‘research data’ in a study and what ‘service user involvement’ is? [73]. In community engagement/participation, a key finding was that peer models appeared to be successful when conducted within a theoretical model like human-centred design or action research that supported ongoing commitment to collaboration, co-learning, mutual leadership and shared decision-making as peers were more likely to feel valued when considered equal members of the project team [84].

## Equality and diversity

Across the review papers there was no mention of any named or unnamed theory relating to equality and diversity. Nineteen of the 31 papers on equality and diversity were identified by review authors as providing evidence for lack of equality and diversity as barriers or enablers. We identified the remaining 11 papers as reporting equality and diversity issues, but not as either barriers or enablers [see Table 3 for key evidence on equality and diversity]. This suggests studies in the systematic reviews are not identifying equality and diversity issues as barriers and enablers to involvement.

Key themes where barriers and enablers were identified included those to do with personal characteristics and involvement, where ethnicity, age and disease might lead to discrimination, and therefore lower opportunities for involvement [65]. In the community engagement and participation literature, barriers identified included a racial divide leading to dissatisfaction amongst predominantly African-American community members who were not being paid and mainly white researchers who were being paid; gender inequality was seen as leading to skills being devalued in male-dominant settings and that the socio-economic status of a community such as poor education, unemployment and poverty affected rates of community participation [81]. There are challenges to gather a well-balanced group that represent different ages, socioeconomic and ethnic groups and which consists of patients in different stages of the disease [60]. In safety, there is inconclusive evidence with respect to age, gender or education to predict patients’ willingness or ability to engage with their own safety [44, 48]. In Patient Service User Research (PSUR), an enabler in involvement processes was that it should consist of individuals or communities for whom the outcomes are of interest [78].

In the author-identified evidence on equality and diversity, in involvement in treatment decision-making, younger age was seen as a facilitator and older age and non-English primary language a barrier in patient safety [46]. Criteria used for patient selection (i.e., representation, demographics) in different studies varied [60]; only a few reviews reported PPI contributor characteristics [80] and the characteristics of those who influenced participation decisions in research [75]; participant [outcomes] reported were limited and primarily to do with English-speaking, literate adults and elderly patients [45]. Attributes patients may need to possess to be involved included excellent interpersonal and communication skills, but these could also exclude other groups such as elderly people, people belonging to ethnic minority groups and people for whom English is not their first language [55].

A significant barrier identified concerned the representativeness of consumers and the need to ensure a much broader representation of groups [addressing age, race/ethnicity, socio-economic and wider healthcare experiences] is involved in PPI activities [49, 54, 59, 60]. Service users were identified as having diverse perspectives, experiences, expectations and interests, which needed to be taken into consideration in relation to the purpose, aims and context of any proposed research, where possible with service users themselves [73]. In guideline development, a key barrier identified was in patients following and assessing medical and technical jargon, which led to patients giving little input. This could lead to high selection standards for patient candidates, leading to issues of representation, if only highly educated patient representatives were recruited [58].

More generally, in the evidence identified on equality and diversity, the issue of representation was given prominence [50, 65, 67, 72]. No references were identified in areas like surgical research regarding involving marginalised and minority groups or on the use of PPI to integrate the opinions of marginalised and minority groups [76], including those traditionally excluded [67].

Regarding methods and strategies for promoting PPI and equality and diversity, barriers exist with a disregard for cultural beliefs and language [77], procedures are needed for the process and preparation for involving users in the classroom and to promote partnership, involvement and equality [57]. Use of language, in terms of health literacy and especially with the use of technical terms and accessible dissemination approaches, are needed for individuals with different abilities and preferences, which consider language and terminology according to the target population [78].

Useful approaches identified for working with communities included: reaching out to relevant communities and not expecting them to come to you [69], use of equivalent training opportunities for mental health users as with employees [74], training in the area of equal opportunities and equality and diversity [68], involvement of authority figures in the community was seen as helpful for buy-in [78], working with the community seemed to provide some opportunities for working with a broader diversity of patient groups [41] and use of bilingual researchers and a need to explore culturally appropriate solutions [77]. In studies where patients developed their own care strategies, some of these strategies were considered to enhance care experiences or equitably improve outcomes and had greater impact in more socio-economically marginalised communities [82]. When patient groups assisted in recruitment and retention of research participants among marginalised populations, they produced greater recruitment numbers and retention [82].

Evidence on methods and strategies more generally in the equality and diversity evidence showed: the involvement of service users allowed researchers to have privileged access to a particular population or community groups and working with community leaders, patient networks or voluntary organisations were good approaches to engaging with seldom heard groups [73]. Use of community participation in reaching communities that are marginalised and have poor access to healthcare was highlighted [81]; diverse peer community participation in the planning and implementation of community-level surveys strengthened the communities’ commitment to using their results [82, 84]; various community projects have been used to target disadvantaged groups (youth, isolated families, ethnic minorities), to increase local access to care services [61]; and a conference was described as a means to consult with a range of consumer groups to inform social work training [54]. In medical education, students visited patients living in deprived areas to raise awareness of disease and links to social deprivation and to ensure adequate representation of different patient groups in education [55]. Targeting elderly patients to improve medication safety was seen as a reasonable strategy, as they are a group where comorbidity/poly-pharmacy is common and there is a need to evaluate the ability of other vulnerable groups (e.g., those with communication difficulties, low health literacy or understanding), to be involved [45]. Strategies to avoid stigmatisation and discrimination were not always found to be effective [53]. Evidence also suggests key principles can be used to optimise PPI best practice, which include the need to accommodate individual and collective needs to ensure inclusivity [69].

A further theme was connected to power inequalities in involvement processes. Barriers highlighted included issues where communities often worked in partnerships with statutory or health authorities where the latter held power [81] and with issues of equality in partnership sharing [66]. Therefore, communities perceived participation as tokenistic [81]. A common concern of researchers and patients was that patient engagement may become tokenistic (a false appearance of inclusiveness), resulting in a devaluated patient input [75]. There is a need for researchers to see Patient and Service User Researchers (PSURs) as equal partners and to consider them as a reliable component of the team rather than simply an additional variable or complication [78]. There are also concerns about inequality in the interactions between PSURs themselves [78] and professionals’ resistance to patients’ participation [59]. Wider reviewer evidence on equality and diversity described concerns to do with the wider involvement of all staff, issues of inequality of power between service users and educationalists and that attempts to redress power differentials can be impacted by cultural barriers in an organisation [53]. Potential-related issues of power, privilege, discrimination, financial inequities, accessibility, communication, distrust and cynicism, can also contribute to peer partnership difficulties [84].

## Discussion

Overall these findings highlight some key aspects underpinning the conduct of PPI across health, social care and patient safety, what facilitates and hinders involvement and how involvement practices can be further developed in the future.

The findings from this review show that despite the very long history of patient and public involvement in the public sector, going back to the 1970s, there are still considerable barriers to involvement in practice. In addressing these issues, these findings suggest there are some key areas that could be addressed to considerably empower patients and the public in the involvement process.

In specific areas such as patient safety, people who were able were willing to be involved. Key barriers for patients were connected to a patients’ health status, which is often age related, and the need for patients to be empowered in dealing with their health condition and diagnosis and support to address patients’ lack of proficiency in the native language. The role of health professionals was identified as very important in empowering patients in the involvement process, through clinicians listening or encouraging patients to ask questions, or participate in specific actions, or nurses’ positive encouragement and support. The avoidance of negative reactions and acceptance of involvement and questioning was very important. At the organisational level, there is a need for organisations to place patients at the centre of safety and for the work environment to support this process, staff involved and partnership working.

Key enablers at the personal level include patients feeling confident and comfortable with error prevention. Relatives appear to play an important role, including in ‘speaking up’ on behalf of family members, in many areas of safety.

The review findings suggest there is a need for involvement practice to be conducted as part of a much wider ‘whole system’ approach, which includes actions at the different levels identified in the findings, e.g., the individual or personal level, team level and organisational level.

The key organisational components of involvement practice that need to be addressed (and which have been well documented previously) are set out in the findings sections under barriers and enablers. These highlight the need for a changes in attitudes and for health professional (and other professional) relationships with patients or public members to be conducted in a non-paternalistic way in a spirit of equal partnership, ensuring people do not feel subordinate to clinicians or professional staff and valuing all people, including a diversity of groups. In involvement practice there is a need for clarity of roles and expectations and the provision of knowledge, clear information and communication, which is not only positively associated with involvement in safety at a personal level in people’s own healthcare, but also in enabling the balance of power to be redistributed in collaborative working with health professionals.

A key aspect of the organisational support for involvement is clearly the provision of financial reimbursement for lay people’s time and wider organisational resources to support the time and costs of involvement processes. General support for patient and public involvement was seen as crucial, which included emotional and practical support as well as training, knowledge and skills development and the exploration of wider methods and community approaches for recruitment and involvement in enabling people to participate. In addressing power dynamics, there is a need to address issues with professionals relinquishing power and tokenism, to ensure those involved are included in all stages of the process and can influence decisions at a personal and organisational level.

Only 19 papers (out of the 31 papers that mentioned equality and diversity) identified equality and diversity issues as barriers and enablers to involvement. This is an area that needs further investigation in the research literature. Areas that were seen as important to address in the review papers concerned how personal characteristics (e.g., age, ethnicity, disease, gender, socio-economic status) could lead to discrimination in involvement processes. In patient safety, the evidence was inconclusive with respect to age, gender or education to claim these factors were consistent in predicting patients’ willingness or ability to engage with safety. Commonly people who were able were willing to be involved. Important barriers affecting ability are illness, which is often age related, and ability to communicate in the native language. There is a need for targeted approaches to involve specific groups, including marginalised groups such as those with low literacy, communication or language difficulties.

The issue of representation was mentioned a number of times, in the sense of the need to ensure a much wider group of patients and the public are involved and particularly those who are marginalised or traditionally excluded. Key barriers in this area include a disregard for cultural beliefs and language, strategies to address health literacy and technical language that excludes groups and methods to promote partnership and inclusivity. Some suggestions that were made about methods that could be used to promote wider involvement particularly focussed on community approaches and use of training, including on equality and diversity and use of care and recruitment strategies by patients or patient groups themselves for widening involvement and participation. Issues of power, inequalities and tokenism in involvement processes both in working with healthcare organisations and in research processes, as well as between lay members were identified.

Generally, the review findings showed that the nature of PPI tended to be atheoretical in its approach, with the area of community engagement and participation the most theory driven area, with a wider focus on both the individual and social structures that limit involvement. In patient safety, the theories seemed to focus more on the individual than the social structures. More generally, there is little theoretical focus on looking at power imbalances. This has considerable implications for the way PPI is carried out in practice.

The findings show that a key theoretical challenge is concept and terminological confusion with approaches to involvement. Understanding different concepts and theories from disparate groups, social movements and policies and practices is important in helping to understand the history, context and contested nature of user involvement across health and social care and different ways to involve people based upon this history and experience. For example, various health social movements and groups [women, Black, Asian and minority ethnic (BAME), those with disabilities, lesbian, gay, bisexual, transgender, queer (LGBTQ), older people, harmed patients’ and those with mental health conditions, etc.] have long challenged the nature of oppressive and harmful services and professionally driven agendas, and this has led to these groups calling for more rights-based, non-discriminatory and equitable approaches to involvement in the provision of services.

Understanding different theoretical traditions can illustrate the underlying values and differentiated nature of involvement, connected to the way that different groups have experienced and been involved in health and social care and the barriers and enablers that different groups face. These different theoretical traditions, such as those highlighted in Table 3 [and particularly under community engagement and participation in health], also illustrate different models that can widen involvement and be used to understand and address power imbalances in involvement processes.

## Strengths and limitations of this study

The strength of this study is that it aimed to critically evaluate and synthesise evidence across a wide range of reviews in health, social care and patient safety. Key to this study was to identify any specific knowledge and theory gaps that could be used to strengthen PPI in patient safety. Quality appraisal of the systematic reviews was assessed using the Assessment of Methodological Quality of Systematic Reviews (AMSTAR 2) [43] tool. This instrument was developed empirically for documenting the methodological quality of systematic reviews, including randomised controlled trials and nonrandomised studies. A limitation of this study was that it did not include looking at the grey literature and the review focussed solely on published peer-reviewed systematic reviews. We retrieved data from information reported by the review authors; therefore, we were limited by the information reported. However, as the focus was on systematic reviews rather than individual studies, there was greater scope to obtain generality in research findings.

## Conclusion

These review findings suggest that a commitment to involvement and partnership working can only be delivered by taking a broader systemic approach to involvement, which considers multiple factors at the individual, team and organisational levels and that addresses the imbalances of power experienced by different groups of patients and the public in the involvement process. This review suggests that addressing equality and diversity is a neglected aspect of PPI. Where there is concern about the lack of marginalised and seldom heard groups, there is little discussion about how to address these concerns. There is clearly a need to explore this area more widely and to look at all aspects of discrimination as covered by the Equality Act of 2010 [85]. The development of theory-driven approaches is also a neglected area, which if developed can lead to a better understanding of the issues involved at an individual, organisational and socio-political level. This understanding can help to guide the process of involvement in more strategic ways across health, social care and patient safety. Key areas for further development in patient safety include the need to explore in more detail how issues of discrimination and inequality between different groups can impact involvement, how theories about the social context and power are relevant and ways to tackle barriers and enablers that are common across different sectors in involvement processes. The longer tradition of involvement and expertise that exists across health and social care about involving patients and the public can provide fertile ground for thinking about ways to try and address these issues in patient safety.

## Supplementary Information

**Additional file 1.** Example Search Strategy-Medline.

## Data Availability

Not applicable.

## Appendix 1. Assessment of methodological quality of the included systematic reviews using the AMSTAR 2 tool

### AMSTAR 2 elements

1. Did the research questions and inclusion criteria for the review include the components of PICO?
2. Did the report of the review contain an explicit statement that the review methods were established prior to the conduct of the review? (critical domain)
3. Did the review authors explain their selection of the study designs for inclusion in the review?
4. Did the review authors use a comprehensive literature search strategy? (critical domain)
5. Did the review authors perform study selection in duplicate?
6. Did the review authors perform data extraction in duplicate?
7. Did the review authors provide a list of excluded studies and justify the exclusions? (critical domain)
8. Did the review authors describe the included studies in adequate detail?
9. (critical domain)
  - Did the review authors use a satisfactory technique for assessing the risk of bias (RoB) in individual studies that were included in the review (RCTs)?
  - Did the review authors use a satisfactory technique for assessing the risk of bias (RoB) in individual studies that were included in the review (NRS)?
10. Did the review authors report on the sources of funding for the included studies?
11. (critical domain)
  - If meta-analysis of RCTs was performed did the authors use appropriate methods for statistical combination of results?
  - If meta-analysis of NRSIs was performed did the authors use appropriate methods for statistical combination of results?
12. If meta-analysis was performed, did the review authors assess the potential impact of RoB in individual studies on the results of the meta-analysis or other evidence synthesis?
13. Did the review authors account for RoB in individual studies when interpreting/discussing the results of the review? (critical domain)
14. Did the review authors provide a satisfactory explanation for, and discussion of, any heterogeneity observed in the results of the review?
15. Did the review authors carry out an adequate investigation of publication bias and discuss its likely impact on the results of the review? = (critical domain)
16. Did the review authors report any potential sources of conflict of interest, including any funding they received for conducting the review?

## Abbreviations

PPI: Patient and Public Involvement
PPE: Public and Patient Experience and Engagement
PRISMA: Preferred Reporting Items for Systematic Reviews and Meta-Analyses
MeSH: Medical Subject Headings
AMSTAR: Assessment of Methodological Quality of Systematic Reviews
PSUR: Patient Service User Research
