# Supplementary material for: Exploring the theory, barriers and enablers for patient and public involvement across health, social care and patient safety: a systematic review of reviews
Source: Health Res Policy Syst. 2021 Jan 20;19:8. doi: 10.1186/s12961-020-00644-3 (PMC7816359; doi:10.1186/s12961-020-00644-3)
Supplement: Supplementary file 1 — Additional file 1. Example Search Strategy-Medline. [file 12961_2020_644_MOESM1_ESM.docx]

**Example Search Strategy- Medline**

| **Searches** | **Results** |
| --- | --- |
| consumer participation/ | 15860 |
| patient participation/ | 22136 |
| or/1-2 | 37690 |
| (patient* adj1 (participat* or involv* or engag* or partnership or partners or collaborat* or consult*)).ab,ti. | 24412 |
| (public adj1 (participat* or involv* or engag* or partnership or partners or collaborat* or consult*)).ab,ti. | 1982 |
| (user* adj1 (participat* or involv* or engag* or partnership or partners or collaborat* or consult*)).ab,ti. | 1416 |
| (service user* adj1 (participat* or involv* or engag* or partnership or partners or collaborat* or consult*)).ab,ti. | 312 |
| (consumer* adj1 (participat* or involv* or engag* or partnership or partners or collaborat* or consult*)).ab,ti. | 895 |
| (lay adj1 (participat* or involv* or engag* or partnership or partners or collaborat* or consult*)).ab,ti. | 141 |
| (citizen* adj1 (participat* or involv* or engag* or partnership or partners or collaborat* or consult*)).ab,ti. | 404 |
| (carer* adj1 (participat* or involv* or engag* or partnership or partners or collaborat* or consult*)).ab,ti. | 281 |
| (caregiver* adj1 (participat* or involv* or engag* or partnership or partners or collaborat* or consult*)).ab,ti. | 844 |
| (customer* adj1 (participat* or involv* or engag* or partnership or partners or collaborat* or consult*)).ab,ti. | 45 |
| (client* adj1 (participat* or involv* or engag* or partnership or partners or collaborat* or consult*)).ab,ti. | 746 |
| (community* adj1 (participat* or involv* or engag* or partnership or partners or collaborat* or consult*)).ab,ti. | 7297 |
| (stakeholder* adj1 (participat* or involv* or engag* or partnership or partners or collaborat* or consult*)).ab,ti. | 2069 |
| ((patient* and public) adj1 (involv* or participat* or engag* or partnership or partners or collaborat* or consult*)).ab,ti. | 6090 |
| (user led or user-led or lay control or user control).ab,ti. | 201 |
| ((representative* or patient representative* or patient advocate* or expert by experience or famil* or relative* or survivor*) adj1 (participat* or involv* or engag* or partnership or partners or collaborat* or consult*)).ab,ti. | 6905 |
| ((patient* or consumer* or citizen* or advisory) adj1 board*).ab,ti. | 1439 |
| ((patient* or consumer* or citizen* or advisory) adj1 group*).ab,ti. | 75334 |
| ((patient* or consumer* or citizen* or advisory) adj1 panel*).ab,ti. | 1215 |
| (citizen* adj1 (jury or juries)).ab,ti. | 64 |
| or/4-23 | 128820 |
| 3 or 24 | 159935 |
| health services research.mp. or exp health services research/ | 151453 |
| social care research.mp. | 28 |
| social service*.mp. | 8561 |
| public health.mp. or exp public health/ | 6876537 |
| psychology.mp. or exp psychology/ | 276350 |
| psychiatry.mp. or exp psychiatry/ | 122746 |
| sociology.mp. or exp sociology/ | 1228269 |
| nursing.mp. or exp nursing/ | 505413 |
| (mental health or mental healthcare).mp. or exp mental health/ | 135802 |
| secondary care.mp. or exp secondary care/ | 4285 |
| tertiary care.mp. or exp tertiary healthcare/ | 33981 |
| or/26-36 | 8022042 |
| Family Practice/ | 64218 |
| Primary Health Care/ | 65710 |
| Physicians, Family/ | 15936 |
| Community Health Services/ | 29975 |
| Community Dentistry/ | 1198 |
| Community Health Nursing/ | 19280 |
| Community Mental Health Services/ | 18322 |
| Community Pharmacy Services/ | 3625 |
| Home Care Services/ | 31334 |
| Community Mental Health Centers/ | 2918 |
| family pract$.tw. | 8627 |
| general practice$.tw. | 34098 |
| community based.tw. | 41862 |
| community care.tw. | 3486 |
| family medicine.tw. | 7419 |
| family physician$.tw. | 11393 |
| primary care.tw. | 81635 |
| (primary health care or primary healthcare).tw. | 18862 |
| family doctor$.tw. | 3837 |
| primary medical care.tw. | 805 |
| general physician$.tw. | 1361 |
| general practitioner$.tw. | 40695 |
| primary care practitioner$.tw. | 1148 |
| (community adj (health or healthcare or health care)).tw. | 16890 |
| primary healthcare team$.tw. | 101 |
| primary health care team$.tw. | 492 |
| primary medical care team$.tw. | 1 |
| practice nurse$.tw. | 4672 |
| practice manager$.tw. | 374 |
| (gpsi or gpwsi).tw. | 30 |
| (practitioner$ adj3 special interest$).tw. | 68 |
| (primary care or primary health care or general practice or family practice or family medicine).nw. | 40736 |
| or/38-69 | 369470 |
| health care.mp. or "Delivery of Health Care"/ | 651145 |
| (health care services or health care delivery).mp. | 18161 |
| 37 or 71 or 72 | 8180235 |
| 70 or 73 | 8253391 |
| patient safety.mp. or Patient Safety/ | 26848 |
| Safety Management/ | 18778 |
| Medication Errors/ or Patient Harm/ or Medical Errors/ | 26909 |
| patient risk.mp. | 2561 |
| Safety/ or safety.mp. | 388412 |
| Adverse event$.mp. | 102273 |
| adverse drug event$.mp. | 2514 |
| incident$.mp. | 96270 |
| error$.mp. | 271931 |
| error*.mp. | 271931 |
| incident*.mp. | 96270 |
| near miss*.mp. | 1578 |
| fall*.mp. | 196678 |
| slip*.mp. | 14834 |
| trip*.mp. | 306863 |
| 75 or 76 or 77 or 78 or 79 or 80 or 81 or 82 or 83 or 84 or 85 or 86 or 87 or 88 or 89 | 1288414 |
| 74 or 90 | 8892947 |
| 25 and 91 | 125154 |
| ((meta or narrative or bibliometric or systematic) and review).ab,ti. | 106442 |
| exp "review"/ or review.ab,ti. | 2552853 |
| 93 or 94 | 2552853 |
| 92 and 93 | 2052 |
